# Feasibility study on image guided patient positioning for stereotactic body radiation therapy of liver malignancies guided by liver motion

**DOI:** 10.1186/s13014-016-0662-2

**Published:** 2016-06-27

**Authors:** Christian Heinz, Sabine Gerum, Philipp Freislederer, Ute Ganswindt, Falk Roeder, Stefanie Corradini, Claus Belka, Maximilian Niyazi

**Affiliations:** Department of Radiation Oncology, LMU Munich, 81377 Munich, Germany

**Keywords:** Stereotactic body radiation therapy, Liver, Patient positioning, Fiducial marker, CBCT

## Abstract

**Background:**

Fiducial markers are the superior method to compensate for interfractional motion in liver SBRT. However this method is invasive and thereby limits its application range. In this retrospective study, the compensation method for the interfractional motion using fiducial markers (gold standard) was compared to a new non-invasive approach, which does rely on the organ motion of the liver and the relative tumor position within this volume.

**Methods:**

We analyzed six patients (3 m, 3f) treated with SBRT in 2014. After fiducial marker implantation, all patients received a treatment CT (free breathing, without abdominal compression) and a 4D-CT (consisting of 10 respiratory phases). For all patients the gross tumor volumes (GTVs), internal target volume (ITV), planning target volume (PTV), internal marker target volumes (IMTVs) and the internal liver target volume (ILTV) were delineated based on the CT and 4D-CT images. CBCT imaging was used for the standard treatment setup based on the fiducial markers. According to the patient coordinates the 3 translational compensation values (*t*_*x*_*, t*_*y*_*, t*_*z*_) for the interfractional motion were calculated by matching the blurred fiducial markers with the corresponding IMTV structures. 4 observers were requested to recalculate the translational compensation values for each CBCT (31) based on the ILTV structures. The differences of the translational compensation values between the IMTV and ILTV approach were analyzed.

**Results:**

The magnitude of the mean absolute 3D registration error with regard to the gold standard overall patients and observers was 0.50 cm ± 0.28 cm. Individual registration errors up to 1.3 cm were observed. There was no significant overall linear correlation between the respiratory motion and the registration error of the ILTV approach.

**Conclusions:**

Two different methods to calculate the translational compensation values for interfractional motion in stereotactic liver therapy were evaluated. The registration accuracy of the ILTV approach is mainly limited by the non-rigid behavior of the liver and the individual registration experience of the observer. The ILTV approach lacks the accuracy that would be desired for stereotactic radiotherapy of the liver.

## Background

Stereotactic body radiation therapy (SBRT) has emerged as an alternative in treatment of hepatocellular carcinoma (HCC) [1–4] and oligometastatic liver disease [5–7] over the past decade. Nevertheless, SBRT of liver malignancies is challenging due to the high biologically effective doses and the uncertainty of the tumor position resulting from a combination of a) intrafractional quasiperiodic motion resulting from the patient’s respiration, b) intrafractional motion (e.g., baseline shifts due to relaxation) and c) interfractional motion resulting from e.g., different fillings of the gastrointestinal tract and patient positioning.

In general, uncertainties resulting from intrafractional motion are minimized (e.g., abdominal compression, breath hold, gating) and compensated by specific margin concepts (e.g., ITV). Interfractional motion, which leads to a systematic shift of the tumor position, is usually managed by image guidance modalities (e.g., CBCT, ultrasound). However, imaging of liver malignancies and hereby the compensation of interfractional motion is challenging.

Regularly, the skeletal anatomy is inadequate for SBRT positioning [8] and the target itself may not be detectable in CT or CBCT. Hence only a surrogate of the exact tumor position can be visualized (e.g., fiducial markers [9]). On the one hand, it has been shown that the implantation of fiducial markers next to the tumor allows a high-precision tumor radiation setup [10, 11], on the other hand the implantation of fiducial markers is naturally an invasive procedure that is not applicable for all patients and thereby limits the application range of SBRT. Furthermore, fiducial markers compromise the image quality of the planning CT [12, 13] and make the tumor delineation more difficult. It is obvious that a different surrogate of the exact tumor position is desirable.

In this retrospective study, the compensation method for the interfractional motion using fiducial markers (gold standard) was compared to a new approach which does not rely on fiducial markers but on the organ motion of the liver and the relative tumor position within the liver volume.

## Methods

### Patients

Starting in 2014 this retrospective study includes 6 patients (3f, 3 m), that suffered from HCC (5/6) and oligometastatic disease (1/6). Prescribed doses and fractionation depended on localization, size of the lesion, mobility and liver function. One patient was not able to complete the radiotherapy course due to an accident resulting in a humerus fracture.

### Preparation and treatment workflow

All patients received a magnetic resonance imaging (MRI) scan and fiducial markers were implanted prior to the planning CT acquisition. Patient positioning for CT imaging and treatment was realized by the use of a vacuum cushion in combination with the WingSTEP (Elekta AB, Sweden). The patients were instructed to breathe freely, and respiration was not restricted by any device. Afterwards, a treatment planning CT and a 4D-CT were acquired. The spatial resolution of the treatment CT (1.074 mm × 1.074 mm × 3.0 mm) was equal to the resolution of the 4D-CT, which consisted of 10 respiratory phases. For each patient an internal target volume (ITV) was delineated containing the gross tumor volume (GTV) outlines of the 10 respiratory phases enlarged by an isotropic margin of 6 mm. Furthermore each fiducial marker was delineated in all respiratory phases and a covering volume (hull) without any additional margin was generated, hereafter referred to as “internal marker target volume” (IMTV). In analogy to the IMTV a second structure set has been created for this study, including only an “internal liver target volume” (ILTV). For the daily treatment, all patients were setup by using CBCT imaging. Due to the slow temporal resolution of the CBCT, fiducial markers were blurred over several respiration cycles similar to the contoured IMTVs generated from the patient’s 4D-CT. According to the patient coordinates defined in the DICOM standard, the 3 translational compensation values (*t*_*x*_*, t*_*y*_*, t*_*z*_) for the interfractional motion were calculated by matching the blurred fiducial markers with the corresponding IMTV structures. Afterwards the compensation values were sent to a robotic couch to correct the daily patient position.

### Recalculation of the compensation values using different approaches

In this retrospective study, 4 observers were requested to recalculate the translational compensation values. The virtual patient setup was done in MOSAIQ (Elekta AB, Sweden), using the image registration module. Table 1 gives an overview of the patient data.

**Table 1:** Overview of the analyzed patient data

Patient		A	B	C	D	E	F
Tumor type		Olig. met.	HCC	HCC	HCC	HCC	HCC
Tumor localization		Seg. VI	Seg. VIII	Seg. II	Seg. I	Seg. V/VIII	Seg. I + VIII
[GTV]	= cc	3.5	16.6	23.9	72.7	12.9	1.4
[ITV]	= cc	14.1	48.1	55.6	133.2	32.5	3.1
[PTV]	= cc	43.7	109.0	115.1	230.0	75.5	20.9
# fiducial markers		3	2	1	1	1	3
# available CBCTs		7	2	9	5	4	4
Data sets		1–7	8–9	10–18	19–23	24–27	28–31

### Patient setup using fiducial markers

**Fig. 1:**
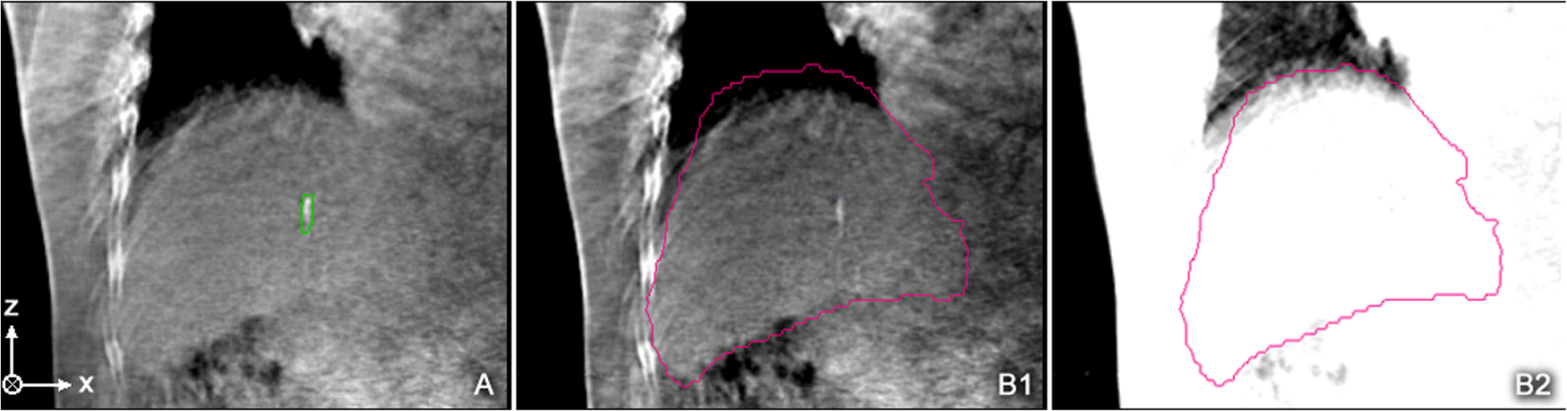
**a** example of a registration based on fiducial markers (*green*); **b** Example of a registration based on the organ motion of the liver (*red*) displayed at different window/level settings

The first method to recalculate the translational compensation values followed a workflow similar to the method used for the daily treatment setup. For each available CBCT a coarse rigid preregistration has been calculated, based on the gray values in CBCT and the initial planning CT. Due to the differences in the daily patient anatomy a second registration step was required. For that reason a manual rigid registration was applied with a strong focus to optimize the correlation between the blurred fiducial markers in the CBCT and the corresponding IMTV structures (see Fig 1a). For each CBCT the resulting translational compensation values were calculated once and served as a gold standard for the second approach.

### Patient setup using organ motion of the liver

The second method also makes use of a coarse rigid preregistration based on the image intensities. But the final manual registration step relies on the ILTV structure that was generated for each patient. Not only the fiducial markers but also the whole liver volume appears to be blurred in the daily CBCT. Therefore the 4 observers were requested to optimize the correlation between the blurred liver volume and the ILTV structure generated from the patient’s 4D-CT (see Fig 1b). Again, the resulting translational compensation components were recorded for each CBCT.

## Results

**Fig. 2:**
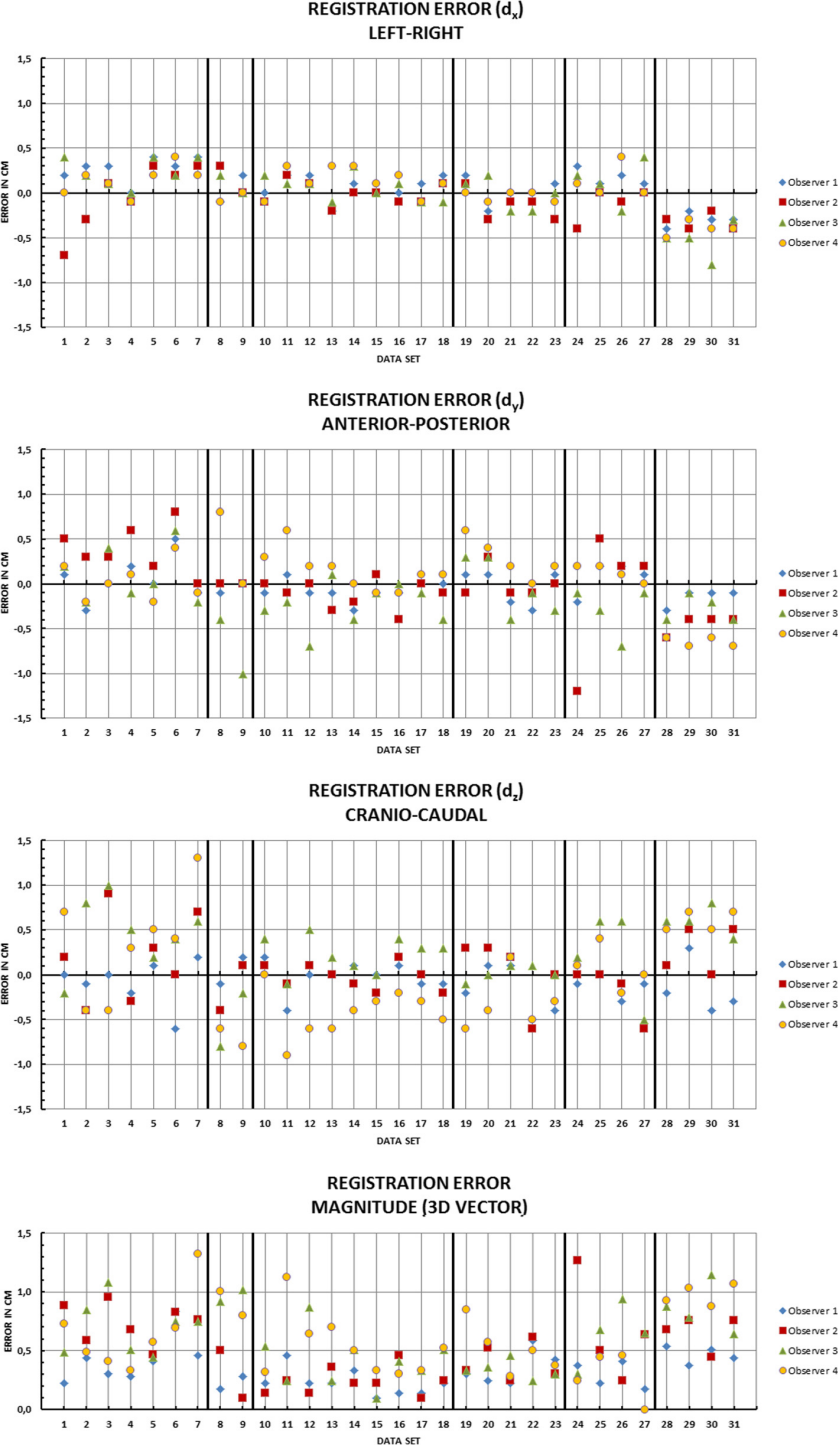
Components and magnitude of all calculated registration errors for all data sets and all observers

In total, 31 different CBCT data sets from 6 different patients were registered by 4 observers using the second approach. As a result of each registration the difference (registration error) of the translational compensation values between the actual registration and the gold standard has been calculated. All values were recorded in cm. Thereby the *d*_*x*_ component denotes the left-right, the *d*_*y*_ component denotes the anterior-posterior and the *d*_*z*_ component denotes the superior-inferior deviation between the actual registration and the registration based on the fiducial markers. Besides, an overall deviation magnitude from all three components was calculated (see Fig 2).

The smallest standard deviation of the registration error over all patients and observers can be observed in the *d*_*x*_ component (0.24 cm), followed by standard deviation of the *d*_*y*_ (0.33 cm) and the *d*_*z*_ component (0.41 cm). The magnitude of the mean absolute 3D registration error over all patients and observers was 0.50 cm ± 0.28 cm. Although the standard deviations of a single component imply a small registration error, individual registration errors of up to 1.3 cm were observed. The evaluation of the data by the individual observers is shown in Table 2.

**Table 2:** Magnitudes of the 3D registration error by the individual observers

Patient	A	B	C	D	E	F
[Magnitude of registration error]	cm	cm	cm	cm	cm	cm
Observer 1	0.42 ± 0.19	0.23 ± 0.05	0.23 ± 0.10	0.36 ± 0.13	0.30 ± 0.10	0.46 ± 0.06
Observer 2	0.74 ± 0.16	0.30 ± 0.20	0.24 ± 0.11	0.40 ± 0.14	0.66 ± 0.38	0.66 ± 0.13
Observer 3	0.70 ± 0.21	0.97 ± 0.05	0.42 ± 0.21	0.34 ± 0.07	0.64 ± 0.23	0.86 ± 0.19
Observer 4	0.65 ± 0.30	0.90 ± 0.10	0.53 ± 0.25	0.52 ± 0.19	0.29 ± 0.19	0.98 ± 0.08

The *d*_*x*_, *d*_*y*_ and *d*_*z*_ components of the registration errors increase in the named order. Hence a relation between the magnitude of respiratory induced motion and the components of the registration error was assumed. From [14] it is known that the organ motion of the liver in the superior-inferior direction is bigger than in the other directions. As a consequence the uncertainty of registration in that direction should also be higher than in the other directions. To check for a correlation between the respiratory motion and the registration errors for each fiducial marker the trajectory of its center of mass (COM) was calculated from the 4D-CT data (Table 3). If there is a correlation between respiratory induced motion and the registration error, it will be reasonable to reduce the respiratory motion by additional actions (e.g., abdominal compression).

**Table 3:** Respiratory induced motion of fiducial markers calculated from 4D-CT

Patient	A	B	C	D	E	F
[Magnitude of motion]	cm	cm	cm	cm	cm	cm
left-right	0.19	0.17	0.16	0.16	0.13	0.08
anterior-posterior	0.46	0.86	0.51	0.19	0.53	0.52
superior-inferior	1.45	1.67	0.79	0.74	1.15	1.52

**Fig. 3:**
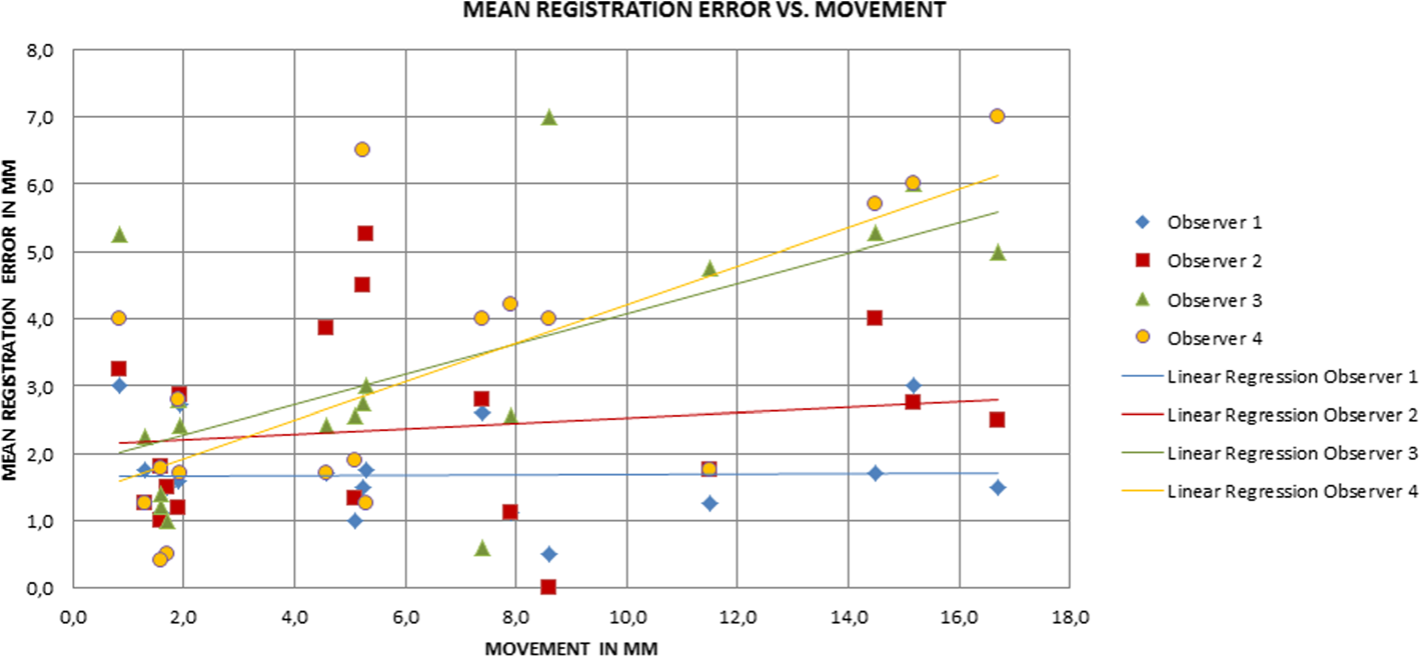
Plot of component wise registration error magnitudes against the movement of the markers (COM) in the components direction

Figure 3 shows the error magnitude of each component plotted against the movement of the fiducial markers (COM) in the same direction. The plot does not show a significant overall linear correlation between the respiratory motion and the registration error. The coefficients of determination (R^2^) are low and vary between different observers (0.0003–0.4929).

## Discussion

Liver SBRT is a highly effective locally ablative technique and may be applied non-invasively. Due to quasiperiodic motion of the liver and its partially non-rigid behavior, fiducial markers are regarded as gold standard when delivering high radiation dose to malignant liver lesions. Within this study, we examined whether fiducial marker implantation was really necessary to account for all different modes of motion. A soft-tissue approach was chosen employing a liver ITV concept (ILTV) being coregistered to a CBCT blurred liver anatomy; if the liver was comparable to a rigid body, this should result in comparable results to the implanted fiducial markers.

We included 31 different datasets from 6 patients in this study and found that the accuracy of the motion based patient setup is limited to 3D registration errors of 0.23 ± 0.05 cm up to 0.98 ± 0.08 cm depending on a specific patient and observer. The largest registration errors were observed in the cranio-caudal direction which is the major direction of liver motion. A componentwise comparison of the registration errors and the corresponding motion extents led to the assumption of a correlation between the motion extent and the registration error. However, this assumption could not be proved since only poor coefficients of determination were found for a linear regression. Therefore it is questionable whether abdominal compression is able to improve the registration results in an organ motion based patient setup significantly.

In addition to the respiratory motion, the low image contrast of soft-tissues in CBCT makes it difficult to correct the patient’s position using the organ motion based patient setup. Hence, the experience of the individual observer has been identified as a main parameter of the registration accuracy.

In a similar study [15], it has been shown, that the soft-tissue-based image guidance with consideration of the 4D breathing motion (organ motion based patient setup) is able to increase the accuracy of treatment compared with stereotactic positioning or image guided radiotherapy without 4D imaging. However, in comparison to the fiducial marker based setup (IMTV), which is supposed to be the superior technique to predict the tumor position compared to other surrogates [11, 16], the registration errors observed in this study imply that the organ motion based patient setup (ILTV) requires adapted safety margins larger than the ones used in a fiducial marker setup.

In general the accuracy of the organ motion based patient setup is limited by the fact that the liver itself does not behave like a rigid object, but is deformed under the influence of respiration. At least three different types of motion were identified in [14], for singular points in the liver volume. For this reason a singular rigid registration of the whole liver will result in different, unknown registration errors depending on the location inside the liver. Above all we found a good agreement of the motion extents of different spots inside the liver compared to [14].

## Conclusions

A new method to compensate for interfractional motion, based on the organ motion of the liver, was compared to the patient setup using fiducial markers. The latter method served as gold standard. It has been shown that a rigid registration based on the organ motion of the liver lacks the accuracy that would be desired for stereotactic radiotherapy of the liver. The registration accuracy is mainly limited by the non-rigid behavior of the liver and the individual registration experience of the observer. Whenever possible, the preferred method to setup the patient and correct for interfractional changes or motion is to place fiducial markers next to the target location and coregister the estimated IMTV to the daily CBCT data.

## Ethics approval and consent to participate

This retrospective study was exempt from requiring ethics approval. Bavarian state law (Bayrisches Krankenhausgesetz/Bavarian Hospital Law §27 paragraph 4) allows the use of patient data for research, provided that any person’s related data are kept anonymous.

## Consent for publication

Not applicable.

## Availability of data and materials

The presented data is summarized in this paper. The complete datasets can be retrieved from the authors upon formal request from interested readers.

## Abbreviations

4D-CT, 4-dimensional computed tomography; CBCT, cone beam computed tomography; COM, centre of mass; CT, computed tomography; GTV, gross tumor volume; HCC, hepatocellular carcinoma; ILTV, internal liver target volume; IMTV, internal marker target volume; ITV, internal target volume; PTV, planning target volume; SBRT, stereotactic body radiation therapy.
